# Teaching About Anti-racism Using a Trauma-Informed Medical Education Framework

**DOI:** 10.1007/s40670-024-02147-0

**Published:** 2024-09-20

**Authors:** Manasa S. Ayyala, Amar D. Desai, Ondrea McKay, Maria Soto-Greene, Michelle DallaPiazza

**Affiliations:** 1 Department of Medicine, Rutgers New Jersey Medical School, Newark, NJ 07103 USA; 2 Rutgers New Jersey Medical School, Newark, NJ USA; 3 Office of Student Affairs, Rutgers New Jersey Medical School, Newark, NJ USA; 4 Office for Diversity and Community Engagement, Rutgers New Jersey Medical School, Newark, NJ USA

**Keywords:** Trauma-informed medical education, Structural racism, Anti-racism, Dialogue

## Abstract

**Background:**

Creating spaces that prioritize trauma-informed medical education (TIME) can support productive learning around anti-racism.

**Activity:**

A pilot virtual-guided book dialogue workshop on anti-racism for 4th year medical students using the TIME framework.

**Results:**

In pre- and post-surveys, students reported high satisfaction with the content and virtual format with significant increases in confidence in achieving the learning objectives and in comfort levels.

**Discussion:**

A TIME approach was effective in increasing confidence and comfort levels in dialoguing about anti-racism. Additional inquiry to provide insight on the benefits of virtual learning for anti-racism content is needed.

**Supplementary Information:**

The online version contains supplementary material available at 10.1007/s40670-024-02147-0.

## Background

Given the profound impact of racism on health, academic medicine must move forward to develop core competencies grounded in anti-racism—deconstructing the effect of structural racism on health outcomes, studying new innovations, and calling to action to build more equitable systems [1]. In this context, learners frequently have varying levels of comfort in discussing racism. Conversations can be challenging both for students who struggle to identify racism and for those who are exposed to racism as a part of their daily lived experiences [2].

Trauma-informed medical education (TIME) guides us to *realize* the widespread impact of racial trauma, particularly among underrepresented in medicine (URIM) students, *recognize* and prepare for trauma responses, *respond* with policies and practices that embody anti-racist values and support safety, and *resist* retraumatizing those who may have a significant personal trauma experience [3, 4]. The importance of incorporating trauma-informed care (TIC) principles to mitigate consequences and potential for repeat trauma should be prioritized in medical education [4]. TIME highlights an educational lens through which a TIC framework can be used with learners in the medical education environment. Using a TIME lens, a focus on dialogue—the process of using empathy and curiosity to explore and develop a deeper understanding of an issue—can provide students with specific skills with which to approach conversations about racism in a psychologically safer space [5–7]. This paper describes the design, implementation, and evaluation of a novel pilot educational intervention for the 4th year graduating medical school class of 2021 exploring structural racism with their peers using a TIME framework. Using pre-assigned chapters from the book *So You Want to Talk about Race*, by Ijeoma Oluo [8], this virtual workshop explored how to create safer spaces for engaging in dialogue about racism. Our goal for the workshop was for students to develop greater confidence in engaging in conversations around structural racism as they progressed through their medical training.

## Activity

### Workshop Description

The workshop was a part of a longitudinal curriculum designed, developed, and implemented using Kern’s six-step approach to curriculum development in medical education [9]. We scheduled the mandatory workshop in the 2020–2021 academic year for 4th year medical students as a part of their transition to residency course in April 2021, just prior to graduation. Students had previously participated in educational sessions throughout the first 3 years of medical school on health equity, including an introductory session on racism and health and one on addressing microaggressions [10] which helped identify the need for this workshop focused on TIME.

Table 1 summarizes the learning objectives and key TIME elements of the workshop. The short didactics reviewed key definitions, described the levels of racism and their effects on health, and explored ten important communal agreements for dialogue (Online Resource 1). The speakers for the didactics also spoke to their intersectional identities and the implications these identities have had on their educational and clinical work.

**Table 1:** Learning objectives incorporating TIME principles [4] and key elements of the virtual workshop

Learning objectives incorporating TIME principles	Additional TIME principles employed
Define racism and anti-racism (Realize) Describe the levels of racism (Recognize) Distinguish debate, discussion, conversation, and dialogue (Respond) Engage in dialogue about anti-racism (Resist re-traumatization)	Transparency: During the didactics, faculty spoke about their positionality and life experiences in relationship to the topic Peer support and collaboration: Students self-facilitated the dialogue in small groups of 5–6 Safety and empowerment: Elements of safety and modeling of productive dialogue were emphasized in the communal agreements

We centered the small group dialogue on the “Introduction” and first three chapters of *So You Want to Talk about Race*. We chose this book because of (1) the accessible narrative; (2) the definition of racism in the context of systems of power and how this can be extrapolated to health care; and (3) the practical advice for talking about race and racism. We provided students with paper or electronic copies of the book so that they could complete the assigned reading in advance of the workshop. The didactic and small group components used a virtual format with break-out rooms.

The small groups were composed of five to six students and were randomly assigned by the virtual platform; each group chose a recorder to summarize key take-home points on a detailed worksheet (Online Resource 2), as well as a reporter to report back to the larger group during the debriefing session. We intentionally designed the small group to be facilitated by the students themselves, so that there would be less pressure to agree with a faculty member within a perceived power imbalance. After the small group, the speakers and students then reconvened as a large group to debrief and brainstorm about how participants can engage in meaningful dialogue after medical school.

## Results

We asked students to complete pre- and post-surveys using a QR code to link to a survey manager. The pre-survey asked students to create a unique anonymous identifier as well as basic demographic information (race and gender identity), specialty choice, a rating of their confidence in achieving each of the learning objectives, one item assessing how much they value engaging in dialogue, and one assessing their comfort levels in having conversations about racism. We rated each of the confidence and the value/comfort level questions on a 5-point Likert scale, with 1 indicating “hardly at all” and 5 indicating “to a very high degree.” The post-survey included the same items related to confidence and value/comfort level, asked the students to rate their satisfaction with each element of the workshop, and prompted text responses about strengths and suggestions for improvement. We matched pre- and post-surveys and excluded those that could not be matched or were incomplete.

### Data Analysis

We performed univariable and multivariable analyses using Microsoft Excel (Microsoft, Seattle, WA) and SAS Software (SAS Studio Release 3.8, Cary, NC). We compared changes to the confidence and value/comfort level items among specialty choice and demographic groups using both paired *t*-tests and chi-squared tests. Multivariable linear regression was used to assess demographic and specialty choice predictors of improvement from the intervention. This study was approved by a Rutgers University Institutional Review Board.

### Outcomes

In total, 183 students participated in the workshop. Of 167 who completed either a pre- or post-survey, 135 (74%) had matched pre- and post-survey responses and were included in the study. Table 2 summarizes the demographic information and specialty choices for the participating students.

**Table 2:** Demographics and specialty choice of the 135 respondents with matched pre- and post-survey responses

Demographic	Number (percentage)
Gender	
Cisgender woman	64 (48%)
Cisgender man	68 (50%)
Gender non-binary, other, choose not to answer	3 (2%)
Race/ethnicity	
American Indian/Alaskan Native	0 (0%)
Asian	52 (39%)
Black/African American	12 (9%)
Hispanic/Latino/Latinx	12 (9%)
White	52 (39%)
Multi-ethnic/other	7 (5%)
Native Hawaiian/Pacific Islander	0 (0%)
Choose not to answer	0 (0%)
Specialty choice	
Primary care	25 (19%)
Internal medicine/pediatrics subspecialty	29 (22%)
Psychiatry	7 (5%)
Emergency medicine	7 (5%)
Neurology	2 (2%)
Obstetrics/gynecology	8 (6%)
General surgery	8 (6%)
Surgical subspecialty	15 (11%)
Physical medicine and rehab	7 (5%)
Pathology	1 (1%)
Anesthesiology	14 (11%)
Choose not to answer/other	11 (8%)

**Fig. 1:**
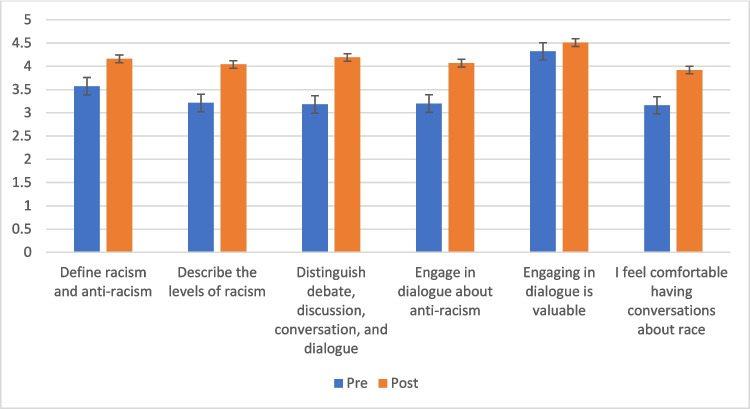
Mean pre- and post-survey responses, *N* = 135; *p* =  < 0.05 for all workshop participants

At baseline and in the post-survey, students who identified as Black or African American reported placing a higher value on the role of dialogue and had higher comfort levels having conversations about race compared to other racial groups (*p* < 0.05). For all participants, we found significant increases in both the confidence in achieving the learning objectives and comfort levels in dialoguing about racism (Fig. 1). When stratifying by self-identified gender, race, or specialty choice, there were no significant or consistent differences in a linear regression analysis. Students reported a high satisfaction with the overall content (mean score on 5-point Likert scale 4.33), and the virtual format of the session (mean 4.22), as well as with each of the components of the workshop, including the didactics (mean 4.16), the small group component (mean 4.27), the peer facilitation (mean 4.25), and the book chapters (mean 4.31).

Suggested improvements included reading more chapters or additional texts (*n* = 8), and incorporating more sessions like this in the overall curriculum or earlier in training (*n* = 5). Feedback on the strengths of the workshop focused on the value of being able to dialogue with peers in a small group setting and/or the quality of the worksheet guiding questions (*n* = 23) and the high quality of the book (*n* = 10). Several commented that the didactic session setting up the communal agreements was very helpful for setting the stage (*n* = 10), and that it was beneficial not to have faculty present in the small groups so that students could speak more freely (*n* = 4).

## Discussion

This workshop was needed at a time when many of our students craved both the space and skills to talk about racism and its impact on health. Using a TIME approach can be effective in attending to spaces of potential discomfort, with the understanding these can also be spaces of growth. Based on feedback comments, the setting of a worksheet-guided, peer-facilitated small group created a learning environment that encouraged dialogue.

Interestingly, while face-to-face small group sessions have many benefits, we found that our students were largely satisfied with the virtual format. Furthermore, we noted a few advantages to the virtual format, including the ability to create smaller groups with a large class size since physical space constraints were eliminated, perhaps also leading to a greater sense of comfort to share ideas. Additional inquiry into virtual spaces for dialogue may help to provide deeper insight for the benefits and limitations of virtual learning for anti-racism content.

Our evaluation is also limited by the small sample size and lack of long-term outcomes related to behavior change. As a pilot innovation, however, it does show promising results with respect to the students’ perceived learning and comfort levels. As a result, we now view the TIME framework as foundational for a curriculum in health equity, and have incorporated content on dialogue skills into our longitudinal health equity content. Limiting this education exclusively to students can have important drawbacks. In order to create spaces of greater psychological safety across multiple learning environments, health equity training at all levels will be critical, particularly as we strive to continue to attract and retain URIM students, trainees, and faculty.

## Conclusion

The marked impact of racial inequities in health and wealth has made conversations about the impact of racism on health imperative in medical education. The pilot workshop we describe in this article using TIME principles increased comfort levels for students to learn core anti-racism concepts. Ultimately, continued content development will be vital as academic medical centers take on a greater role in addressing racial health disparities and in upholding anti-racist policies and practices.

## Supplementary Information

Below is the link to the electronic supplementary material.

Supplementary file1 (DOCX 16 KB)

Supplementary file2 (DOCX 17 KB)

## Data Availability

The data generated analyzed for this study are available on request from the corresponding author.
