# Supplementary material for: Teaching About Anti-racism Using a Trauma-Informed Medical Education Framework
Source: Med Sci Educ. 2024 Sep 20;35(1):33–6. doi: 10.1007/s40670-024-02147-0 (PMC11933480; doi:10.1007/s40670-024-02147-0)
Supplement: Supplementary file 1 — Supplementary file1 (DOCX 16 KB) [file 40670_2024_2147_MOESM1_ESM.docx]

**Supplementary Table 1**: The ten communal agreements of dialogue

| **Agreement** | **Additional points / recommendations** |
| --- | --- |
| 1. Listen actively | Remain present |
| 1. Remain curious | Be open to new paradigms and perspectives, and approach learning with humility |
| 1. Ask for clarifications when needed | Respectfully challenge expressed beliefs when appropriate |
| 1. Speak from your own experience | Avoid making generalizations based on your own experience  Leave space for others to speak to their own experiences |
| 1. Avoid making assumptions about the beliefs, values, and motives of others | Remain calm and non-judgmental  When prompted, name and talk about your emotions and why you have them |
| 1. Refrain from personal attacks | Focus on the ideas/behaviors, not the person |
| 1. Consider the intent and the impact of words and actions | Understand that this is especially important when you find yourself in a majority or a socially-dominant position  Freely and genuinely apologize when impact does not align with the intent |
| 1. Reveal only what you feel comfortable revealing and practice self-preservation | Avoid pressuring others to participate if they feel uncomfortable  It is not the job of URIM individuals to do all of the work  Appreciate that these topics are deeply intertwined with identity and trauma experiences, and can be intensely personal  Set boundaries, and find avenues for self-care and community |
| 1. There is no one “right” answer | No one has all of the answers  This is a life-long journey  The best solutions will require collective action |
| 1. The goal is not always to agree, but to gain a deeper understanding | Even when we have differing opinions, gaining a deeper understanding and creating shared narratives brings us closer together in a shared goal of serving our patients  New insights can help you in the work you  do to dismantle racism in medicine |
