# Supplementary material for: Teaching About Anti-racism Using a Trauma-Informed Medical Education Framework
Source: Med Sci Educ. 2024 Sep 20;35(1):33–6. doi: 10.1007/s40670-024-02147-0 (PMC11933480; doi:10.1007/s40670-024-02147-0)
Supplement: Supplementary file 2 — Supplementary file2 (DOCX 17 KB) [file 40670_2024_2147_MOESM2_ESM.docx]

**Supplementary Table 2:** Online peer-facilitated worksheet for small group dialogue using the book, *So You Want to Talk about Race* by Ijeoma Oluo

| **Directions** | For this small group session, use the following prompts and questions to guide the dialogue.  Choose a student to read each section and pose the questions to the group (this can be a different student for each section).  You will also need to choose a student to serve as the **recorder**. To enhance participation of the recorder, the only section that requires a written response is the very last section, where the group will brainstorm about **three important takeaways**.  Please keep in mind the communal agreements for dialogue reviewed in the lecture portion. |
| --- | --- |
| **Introduction**  15 minutes | In the introduction, the author talks about how she had spent much of her early life not talking, thinking, or feeling about race and racism. Instead, life was spent “trying to get by” in a life that was “busy and hard.”  In many ways, we can draw parallels with our medical training, where we spend our time focusing on essential questions related to diagnosing, examining, and treating patients, and seeking answers to meaningful clinical and research questions—important pursuits in a busy and demanding career.  However, in this space we also often fail to challenge our assumptions or critically appraise the racial disparities we see are all around us. As one example, medicine as a whole rarely questions or addresses why under-resourced hospitals are predominantly located in communities of color, or why physicians in academic medical centers are rarely representative of the patient population, but rather accepts these conditions as the norm.  The author also explores how she reached a moment when she could not stay silent any longer.   - As future leaders in healthcare, what is the role of physicians to dialogue about race and racism in medicine? - During the past year, what societal/current events with respect to race have had an impact on you personally? How have they affected you? What was the response of your loved ones and communities? - In the context of the events of the last year, how has the narrative around race in society and medicine shifted? How have these events and shifting narratives influenced your views or awareness about racism? |
| **Chapter 1: Is it really about race?**  10 minutes | In chapter 1, the author states: “It is about race if a person of color thinks it’s about race. It is about race if it disproportionately or differently affects people of color. It is about race if it fits into a broader pattern of events that disproportionately or differently affects people of color.” She also describes race as something that is alive and interwoven into every aspect of our lives and our institutions. In reflecting on the arguments she makes in this chapter:   - What initial feelings emerged when you considered these three basic rules the author proposes? - What elements of her arguments do you find most compelling in supporting these three rules? - What counter arguments might challenge these rules and does she address these in the chapter? |
| **Chapter 2: What is race?**  10 minutes | In chapter 2, the author defines racism as “any prejudice against someone because of their race, when those views are reinforced by systems of power.”   - What are your thoughts on this systemic definition when compared to the individualized alternative definition “any prejudice against someone because of their race?” How might this definition reframe dialogue about racism? - In reflecting on your clinical experiences, how do you see structural racism manifesting and affecting the lives of patients of color? What are some examples of how structural racism is embedded in medical training and the practice of medicine? - The author suggests that tying racism to its systemic causes and effects can help when dialoguing about the harmful effects of racism. What are some resources you can use or have used in the past to increase your knowledge about the systemic causes and effects of racism on health? |
| **Chapter 3: What if I talk about race wrong?**  15 minutes | In chapter 3, the author starts a section of the chapter with the statement “You’re going to screw this up,” and explores some of the ways that dialogue about race can be difficult and painful.   - Of the basic tips presented to help guide talking about race, which one(s) will you take with you for future conversations? Which might you find to be difficult to implement and why? - What tips will be helpful for you in demonstrating empathy for others, their lived experiences, and their struggles in talking about racism? - What resources can you access for self-care and support? How can you ensure that those resources are available and appropriate to your needs? |
